# Analysis of IVF/ICSI-FET Outcomes in Women With Advanced Endometriosis: Influence on Ovarian Response and Oocyte Competence

**DOI:** 10.3389/fendo.2020.00427

**Published:** 2020-07-17

**Authors:** Anji Li, Jie Zhang, Yanping Kuang, Chaoqin Yu

**Affiliations:** ^1^The First Affiliated Hospital of Guizhou University of Traditional Chinese Medicine, Guiyang, China; ^2^Department of Assisted Reproduction, Shanghai Ninth People's Hospital, Shanghai Jiao Tong University School of Medicine, Shanghai, China; ^3^Department of Gynecology of Traditional Chinese Medicine, Changhai Hospital, Naval Medical University, Shanghai, China

**Keywords:** advanced endometriosis, ovarian response, oocyte competence, *in vitro* fertilization/intracytoplasmic sperm injection, frozen–thawed embryo transfer, endometriosis, IVF/ICSI, FET

## Abstract

**Aims:** To determine the impact of advanced endometriosis (EMS) on *in vitro* fertilization/intracytoplasmic sperm injection and frozen–thawed embryo transfer (IVF/ICSI-FET) outcomes and analyze the influencing factors.

**Methods:** A retrospective study was conducted on sterile women with ovarian endometriomas (OMAs), including patients who underwent laparoscopic cystectomy (*n* = 224, 224 IVF/ICSI cycles, 205 FET cycles) and aspiration (*n* = 139, 139 IVF/ICSI cycles, 148 FET cycles); peritoneal EMS (*n* = 96, 96 IVF/ICSI cycles, 89 FET cycles); and tubal factors (*n* = 360, 360 IVF/ICSI cycles, 474 FET cycles). Our main outcomes included the number of MII oocytes retrieved, fertilization rate, the number of viable embryos, viable embryo rate per oocyte retrieved in oocyte retrieval cycles, and clinical pregnancy rate per transfer, live birth rate per transfer, and cumulative clinical pregnancy rate of this oocyte retrieval cycle in FET cycles. Finally, binary logistic regression analysis was performed to generate a prediction model for cumulative clinical pregnancy.

**Results:** The results showed that significantly fewer MII oocytes retrieved and viable embryos and lower viable embryo rate and cumulative clinical pregnancy rate were observed in women with EMS compared with the control. Women with peritoneal EMS had lower fertilization rate and viable embryo rate per oocyte retrieved than patients with OMA (all *p* < 0.05). However, the pregnancy outcomes were not significantly different between the two phenotypes. The patients who underwent laparoscopic cystectomy had fewer MII oocytes retrieved and viable embryos compared with those with intact endometrioma(s) but no significant difference in pregnancy outcomes between the two types of OMA patients. By binary logistic regression analysis, antral follicle count (AFC) was found to be an independent factor associated with cumulative clinical pregnancy in this oocyte retrieval cycle (odds ratio = 1.054; 95% confidence interval, 1.011–1.100; *p* = 0.014), and the AFC prediction model of cumulative clinical pregnancy was established, with an area under the curve of 0.60.

**Conclusions:** Our data supported that advanced EMS has negative effect on cumulative clinical pregnancy per oocyte retrieval cycle, and AFC is an independent predictor, which is mainly caused by poor ovarian response associated with OMA *per se* or its surgery and the damage of peritoneal EMS to oocyte maturation.

## Introduction

Endometriosis (EMS) is an estrogen-dependent disease in which there is active endometrial-like tissue outside the uterus, primarily on ovaries and the peritoneal peritoneum (1). It affects ~2–10% of women of reproductive age (2) and 30–50% of women experiencing difficulties in fertility (3). The pathogenesis of EMS-associated infertility is poorly elucidated; however, some factors have been studied, including anatomical changes in the reproductive tract, ovulatory dysfunction, disturbed folliculogenesis, and defective implantation (4).

The common choice for the patient with EMS-associated sterility who wish to conceive is assisted reproductive technology (ART). Reports evaluating the impact of EMS on the outcomes of ART seemed controversial, in relation to the focus on different specific outcomes. Endometriosis is related with poor ovarian response (POR), which manifests as the decrease in antral follicle count (AFC) and the number of oocytes retrieved, but irrelevant to the pregnancy/live birth rate in each embryo transfer, from which it was concluded that EMS has no negative impact on ART outcomes (5–7). However, the pregnancy rate per transfer is correlated with endometrial receptivity but ovarian response. Oocyte competence reflects the ability to mature and fertilize. Once oocyte maturation or fertilization is blocked, the number of MII oocytes retrieved and the number of viable embryos are decreased in an oocyte retrieval cycle, respectively. With the same clinical pregnancy rate per transfer, the more embryos that could be transferred, the greater the total chance of clinical pregnancy of this oocyte retrieval cycle. The exact impact of EMS on ART outcomes could be clarified in a study with a large sample size.

Superficial EMS, ovarian endometrioma (OMA), and peritoneal deep infiltrating EMS (DIE) are main phenotypes up to now (8). Different from the other two phenotypes, OMA affects ovaries directly by increasing mechanical stress, rigidity, distorted anatomy to impair blood supply, and innervation of ovaries, associated with diminished oocyte quality and ovarian reserve (9), whereas according to recent reports (10), OMA *per se* is not associated with presentation for infertility. With regard to OMA surgical excision before *in vitro* fertilization/intracytoplasmic sperm injection (IVF/ICSI), some clinicians found that surgeries may reduce ovarian reserves and responsiveness to stimulation (11, 12); nevertheless, other studies have supported the opposite view and found that the serum level of anti–Müllerian hormone (AMH), declining sharply after surgeries, would recover in 6 months (13), and the excision of OMA could improve the success rate of ART in a long-term observation (14). It is not clear whether OMA *per se* or its excision will have a greater impact on the outcome of IVF/ICSI so far. Anatomical abnormality in the peritoneal cavity aside, peritoneal EMS may cause an environment to be less favorable for fertility (15). In the peritoneal fluid of patients with EMS, researchers found increased inflammatory factors (16, 17) and enhanced oxidative stress (18). The spindles play an important role in meiosis and especially sensitive to oxidative stress, which may be one of the causes for oocyte dysmaturity of EMS.

This retrospective study was conducted to (1) examine the impact of EMS on IVF/ICSI frozen–thawed embryo transfer (FET) outcomes, (2) determine whether OMA is the main factor of EMS affecting IVF/ICSI outcomes, and (3) assess the effect of surgical intervention to OMA before IVF/ICSI-FET on pregnancy outcomes. These findings will provide direction for studying the mechanism of EMS-associated infertility and evidence for management of OMA surgical intervention prior to IVF/ICSI.

## Materials and Methods

### Patients

The present analysis included data collected from the Department of Assisted Reproduction of the Ninth People's Hospital of Shanghai Jiaotong University's School of Medicine in a retrospective cohort study. A total of 819 women who had undergone IVF/ICSI treatment from January 1, 2016, to December 31, 2017, were included.

The patients with EMS, stage III–IV according to the revised American Fertility Society (rAFS) classification, consisted of OMA and peritoneal EMS. The patients with OMA included those whose OMAs had been removed by laparoscopic cystectomy and those whose OMAs had been aspirated under transvaginal ultrasound guidance during ovulation monitoring or at the time of oocyte retrieval for histopathologic diagnosis. The patients with peritoneal EMS had been found to have endometriotic lesions in the peritoneal cavity rather than ovaries by laparoscopy, which had been treated. The control group consisted of women with infertility due to tubal factors.

The eligibility criteria were 25–40 years of age, body mass index (BMI) that ranged from 18.5 to 23.9 kg/m^2^, first IVF/ICSI, and normal sperm in the male. The exclusion criteria were chronic anovulation including polycystic ovarian syndrome; adenomyosis diagnosed by laparoscopy, laparotomy, or ultrasound examination or magnetic resonance imaging; presence of hydrosalpinx or of endocrinopathy, cardiovascular disease, dyslipidemia, systemic lupus erythematosus or another rheumatologic disease, human immunodeficiency virus infection, or any active infection; smoking habit; use of hormonal medications or hormonal or non-hormonal anti-inflammatory agents during the 3 months prior to inclusion in this study; and any contraindications to ovarian stimulation treatment.

### Protocol for Controlled Ovarian Stimulation, Embryo Culture, and Frozen–Thawed Embryo Transfer

Each patient was administrated 150–225 IU/day of human menopausal gonadotropin (Anhui Fengyuan Pharmaceutical Co., Hefei, China) and 4 or 10 mg/day of medroxyprogesterone acetate (Beijing ZhongXin Pharmaceutical, Beijing, China) from menstrual cycle day 3 (D3) of menstruation, according to the observed follicular growth by ultrasound and blood test. Ovulation was triggered by human chorionic gonadotropin (hCG) of ~2,000–10,000 IU (Lizhu Pharmaceutical Trading Co., Zhuhai, China) when there were more than three dominant follicles of >18-mm diameter, followed by transvaginal ultrasound-guided oocyte retrieval 36–37 h later. All follicles with diameters >10 mm were punctured, and oocytes were retrieved and *in vitro* fertilized using conventional insemination or ICSI, depending on semen parameters. On the third day after fertilization, embryos were visually estimated and rated according to the criteria of Cummins et al. (19). High-quality embryos (including grade 1 and grade 2 eight-cell embryos) were frozen by vitrification, and the rest were further extended culture until the blastocyst stage, in which only ones with good morphology were cryopreserved on D5 or D6.

Natural FET cycles were used for women with regular menstrual cycles. The women with irregular menstrual cycles were orally administered 2.5–5 mg/day of letrozole from cycle D3 to D7. For patients with thin endometria during ovarian stimulation cycles, oral ethinylestradiol (25 mg two times per day; Xinyi Pharmaceutical Co., Shanghai, China) was recommended from cycle D3 until an endometrial thickness of 8 mm, followed by two tablets of yellow Femoston taken orally [including 2 mg estradiol (E_2_) and 10 mg dydrogesterone per tablet, two times per day; Abbott Healthcare Products B.V., Hoofddorp, the Netherlands] and soft vaginal progesterone capsules (Utrogestan, 200 mg two times per day; Laboratoires Besins International, Ploermel, France). Follicle growth was monitored by serum hormone measurements and transvaginal ultrasound scanning beginning on cycle D10 onward. The administration of hCG to trigger ovulation and the timing of FET were performed as previously described (20). One or two embryos were transferred per FET cycle. The exogenous estrogen and progesterone were continued until 10 weeks of gestation after a positive pregnancy test.

### Data Collection and Study Outcomes

There were three types of data: baseline characteristics, oocyte retrieval cycle data, and FET cycle data. As baseline characteristics, the duration of infertility; primary or secondary infertility; serum follicle-stimulating hormone (FSH), luteinizing hormone (LH), E_2_, and AFC on menstrual cycle D3; and duration and dose of gonadotropin stimulation were recorded.

The number of follicles larger than 14 mm in diameter on the trigger day, number of oocytes retrieved, number of mature oocytes (MII oocyte) retrieved, method of insemination, number of fertilized oocytes, number of normal fertilized oocytes, number of cleavage oocytes, number of 3-day-old (D3) high-quality embryos (grades 1 and 2 eight-cell embryos), number of embryos cultured to blastocyst stage, and number of viable blastocyst were regarded as indicators of follicle development and oocyte performance in oocyte retrieval cycle. Based on the above parameters, we figured out viable blastocyst rate, oocyte retrieval rate, proportion of patients with POR, mature oocyte rate, fertilization rate, normal fertilization rate, cleavage rate, viable embryo rate per oocyte retrieved, and high-quality embryo rate per oocyte retrieved. The viable blastocyst rate was defined as the number of blastocysts with good morphology divided by the number of embryos cultured to blastocyst stage. The diagnosis of POR, according to the Bologna criteria (21), was based on retrieval of three oocytes or fewer and AFC of <5. High-quality embryos included D3 high-quality embryos (grades 1 and 2 eight-cell embryos) and viable blastocysts.

The FET cycle data included the number of embryos transferred, endometrial thickness on the day of transfer, number of clinical pregnancy, number of embryos implanted, number of miscarriage, number of ectopic pregnancy, number of delivery with live birth, term or preterm delivery, and birth weight. Based on the above parameters, we figured out the clinical pregnancy rate per transfer, implantation rate, miscarriage rate, ectopic pregnancy rate, cumulative clinical pregnancy rate per woman, and live birth rate per transfer. The clinical pregnancy was considered in the presence of a gestational sac with or without fetal heart activity, as assessed by ultrasound at 4 weeks of gestation. The implantation rate was defined as the proportion of embryos that developed into gestational sacs in the total transferred embryo. Cumulative clinical pregnancy rate was worked out by the number of patients who ever had a history of clinical pregnancy divided by the number of the patients who had been transferred all embryos from this oocyte retrieval cycle and the patients who had at least one clinical pregnancy from January 1, 2016, to December 31, 2018. Live birth rate per transfer was defined as the number of delivery with live births divided by the number of FET cycles.

### Study Design

This study was composed of three comparisons. In the first comparison, all patients with EMS (EMS group) were compared with patients with infertility due to tubal factors (control group) to evaluate the impact of EMS on IVF/ICSI outcomes. In the second comparison, patients with OMA (OMA group) were compared with patients with peritoneal EMS (peritoneal EMS group) to analyze the difference in IVF/ICSI outcomes between OMA and other phenotypes of EMS. In the third comparison, patients who underwent laparoscopic cystectomy (cystectomy group) were compared with those who underwent OMA aspiration (aspiration group) to assess the impact of surgical intervention to OMA on subsequent IVF/ICSI outcomes.

### Statistical Analysis

By reference to the reports published, the clinical pregnancy rate of ART was 29.6% (22), and that of severe EMS patients was estimated to be 15.7% (23). Excluding 15% lost to follow-up, the power of the study with the sample size (390 in the EMS group and 306 in the control group) was 0.9899, which was computed by PASS 15 software (NCSS, Kaysville, UT, USA). Statistical analyses were conducted using IBM SPSS Statistics 21.0 software (IBM, Almonk, NY, USA). The Kolmogorov–Smirnov test was performed to assess the normality of the distribution in continuous data, which were presented as mean (SD) and assessed using the Mann–Whitney *U*-test for two independent groups. Count data are presented as numbers and percentages and assessed using Pearson χ^2^-test or Fisher exact test. Differences were considered significant if two-sided *p* < 0.05. Binary logistic regression analysis was performed to generate a prediction model for cumulative clinical pregnancy, which was at least one clinical pregnancy before transferring all the embryos from this oocyte retrieval cycle, with the clinical parameters as the independent variables. The clinical parameters included group allocation, age, duration of infertility, BMI, (D3) FSH, LH, E_2_, AFC, number of oocytes retrieved, POR, oocyte retrieval rate, mature oocyte rate, and fertilization rate.

## Results

**Figure 1:**
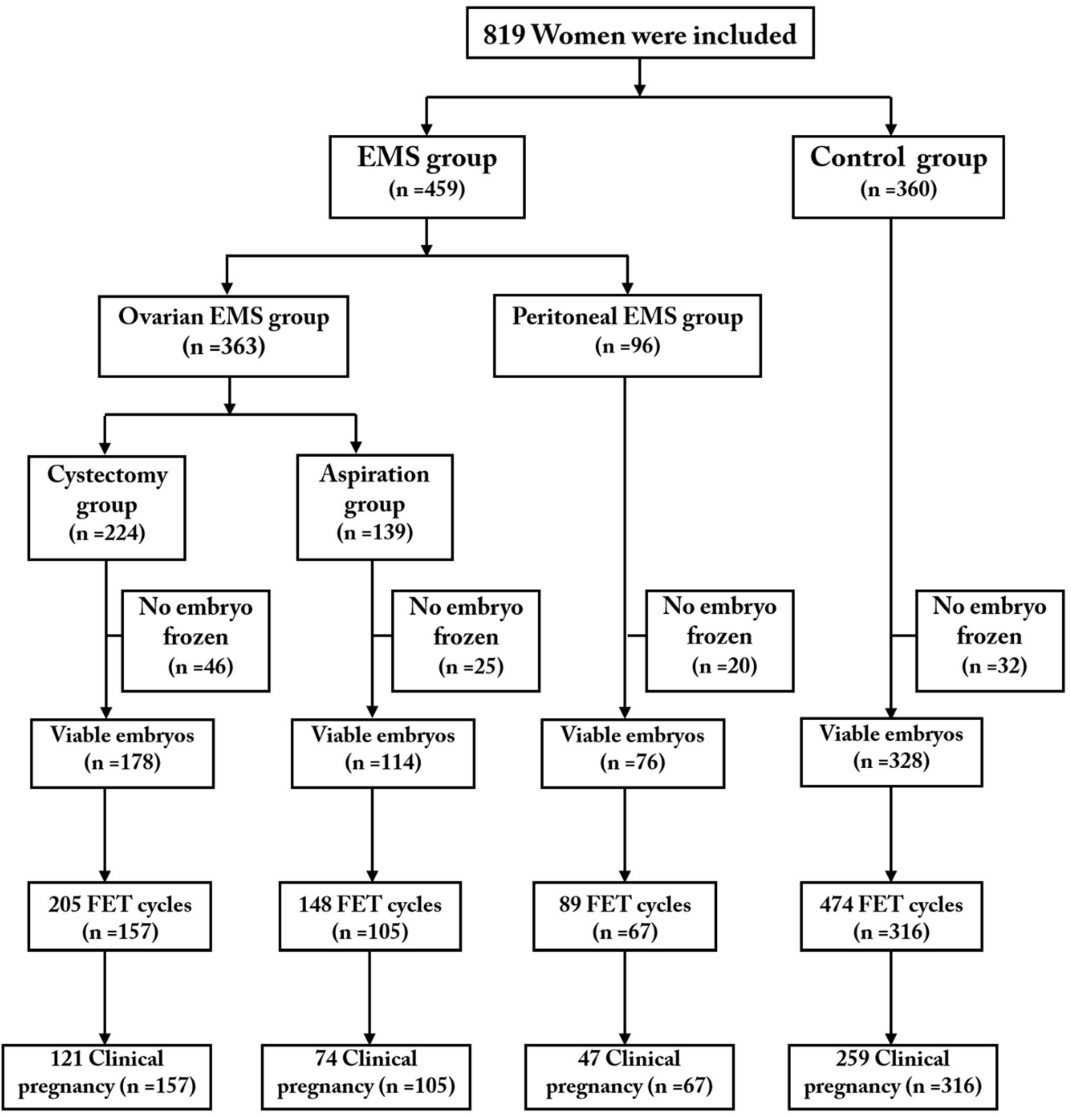
Flowchart for each comparison.

A total of 819 eligible patients (363 patients with OMA including 224 in the cystectomy group and 139 in the aspiration group; 96 patients in the peritoneal EMS group; 360 patients in the control group) were included and completed their first oocyte retrieval cycles. We followed up the patients with FET outcomes to December 31, 2018, 1 year after the last cycle of oocyte retrieval. A total of 645 women (cystectomy group, 157; aspiration group, 105; peritoneal EMS group, 67; control group, 316) completed 916 FET cycles (cystectomy group, 205; aspiration group, 148; peritoneal EMS group, 89; control group, 474). Figure 1 shows a profile summary of the study.

### Patient Characteristics

The baseline characteristics of the patients in the study are shown in Table 1. The patients with EMS had significantly higher percentage of primary infertility (66.0%) and (D3) serum E_2_ level (39.46 ± 34.37 pg/mL) but less AFC (8.00 ± 5.91) compared with the control (34.2%; 33.79 ± 37.85 pg/mL; 10.25 ± 5.64, respectively, all *p* < 0.05). Comparing with the patients with a history of endometrioma, the patients with peritoneal EMS had significantly longer duration of infertility (3.79 ± 2.87 vs. 2.78 ± 2.15 years), lower percentage of primary infertility (57.3 vs. 68.3%), and greater AFC (9.35 ± 7.61 vs. 7.65 ± 5.33) (all *p* < 0.05). In the patients with a history of endometrioma, those who underwent cystectomy had significantly higher (D3) serum FSH level (7.83 ± 11.78 IU/L) compared with those with intact endometrioma(s) (6.12 ± 2.62 IU/L) (*p* = 0.038).

**Table 1:** Characteristics of patients undergoing IVF/ICSI.

**Characteristics**	**EMS group**	**Control group**	***p*1**	**OMA group**	**Peritoneal EMS group**	***p*2**	**Cystectomy group**	**Aspiration group**	***p*3**
No. of patients	459	360		363	96		224	139	
Age (y), mean ± SD	31.46 ± 3.53	31.84 ± 3.8	0.259	31.43 ± 3.57	31.6 ± 3.36	0.662	31.76 ± 3.29	32.50 ± 3.75	0.055
BMI (kg/m^2^), mean ± SD	21.39 ± 1.74	21.19 ± 1.80	0.114	20.92 ± 2.29	20.74 ± 1.79	0.495	21.05 ± 1.75	20.70 ± 2.96	0.214
Duration of infertility (y), mean ± SD	2.99 ± 2.35	2.92 ± 3.00	0.706	2.78 ± 2.15	3.79 ± 2.87	0.002	10.98 ± 6.00	10.71 ± 2.21	0.617
Primary infertility, *n* (%)	303 (66.0)	123 (34.2)	0.000	248 (68.3)	55 (57.3)	0.042	151 (67.4)	73 (52.5)	0.637
Day 3, mean ± SD									
FSH (IU/L)	6.95 ± 8.47	6.17 ± 2.28	0.404	7.17 ± 9.42	6.07 ± 2.21	0.263	7.83 ± 11.78	6.12 ± 2.62	0.038
LH (IU/L)	3.95 ± 4.59	3.67 ± 1.63	0.554	3.99 ± 4.80	3.81 ± 3.66	0.731	4.25 ± 5.98	3.59 ± 1.65	0.209
E_2_ (pg/mL)	39.46 ± 34.37	33.79 ± 37.85	0.026	40.09 ± 34.11	37.04 ± 35.41	0.447	42.74 ± 40.13	35.82 ± 20.49	0.062
AFC, mean ± SD	8.00 ± 5.91	10.25 ± 5.64	0.000	7.65 ± 5.33	9.35 ± 7.61	0.041	7.25 ± 5.20	8.29 ± 5.50	0.068
hMG and MPA duration (d), mean ± SD	10.84 ± 4.51	10.75 ± 1.93	0.699	10.88 ± 4.90	10.72 ± 2.53	0.762	10.98 ± 6.00	10.71 ± 2.21	0.617
hMG dose (IU), mean ± SD	1,567 ± 652	1,629 ± 580	0.151	1,545 ± 668	1,645 ± 586	0.149	1,505 ± 694	1,612 ± 619	0.127
MPA dose (mg), mean ± SD	54 ± 45	51 ± 43	0.351	54 ± 46	54 ± 44	0.962	56 ± 49	50 ± 39	0.161

*p1, EMS group vs. control group; p2, Ovarian EMS group vs. peritoneal EMS group; p3, cystectomy group vs. aspiration group; BMI, body mass index; FSH, follicle-stimulating hormone; LH, luteinizing hormone; E_2_,estrogen; AFC, antral follicle count; hMG, human menopausal gonadotropin; MPA, medroxyprogesterone acetate. The red number highlights the p which was less than 0.05 signifing the statistic difference*.

### Ovarian Response, Follicle Development, and Oocyte Performance in Oocyte Retrieval Cycles

Table 2 shows the oocyte retrieval cycle characteristics of patients in the EMS group and the control group. The EMS group had higher proportion (14.9%) of oocytes inseminated by ICSI comparing with the control group (1.2%; 7.2%, respectively, both *p* < 0.001). The number of follicles with diameter >14 mm, number of oocytes retrieved, number of MII oocytes retrieved, number of D3 high-quality embryos, and the number of viable embryos were significantly fewer and viable blastocyst rate, oocyte retrieval rate, and viable embryo rate per oocyte retrieved were significantly lower in the EMS group compared with those in the control group (all *p* < 0.05). The percentage of patients with POR was 19.2% in the EMS group, in contrast with 7.2% in the control group (*p* < 0.001).

**Table 2:** Oocyte retrieval cycle characteristics of the patients in the EMS group and control group.

**Characteristics**	**EMS group**	**Control group**	***p***
No. of patients	459	360	
No. of oocyte retrieval cycles	459	360	
No. of >14-mm follicles on the trigger day	7.10 ± 5.65	9.35 ± 6.83	0.000
No. of oocytes retrieved	7.88 ± 6.19	11.48 ± 7.65	0.000
No. of MII oocytes retrieved	6.55 ± 5.33	9.59 ± 6.58	0.000
Method of insemination			0.000
IVF, %	85.1 (2,976/3,498)	98.8(3,972/4,019)	
ICSI, %	14.9 (522/3,498)	1.2(47/4,019)	
No. of D3 high-quality embryos	2.61 ± 2.72	3.98 ± 3.50	0.000
No. of embryos cultured to blastocyst stage No. of viable blastocyst Viable blastocyst rate, %	1,334 222 16.6	1,630 376 23.1	0.000
No. of viable embryos, *n*	2.93 ± 2.65	4.57 ± 3.44	0.000
Oocyte retrieval rate, %	68.9 (3,617/5,250)	74.8 (4,134/5,524)	0.000
Poor ovarian response, %	19.2 (88/459)	7.2 (26/360)	0.000
Mature oocyte rate, %	83.0 (3,001/3,617)	83.7 (3,460/4,134)	0.391
Fertilization rate, %	77.5 (2,712/3,498)	78.3 (3,219/4,109)	0.395
Normal fertilization rate, %	73.0 (2,552/3,498)	72.4 (2,973/4,109)	0.557
Cleavage rate, %	97.1 (2,477/2,552)	97.6 (2,901/2,973)	0.234
Viable embryo rate per oocyte retrieved, %	37.2 (1,346/3,617)	39.8 (1,645/4,134)	0.020
High-quality embryo rate per oocyte retrieved, %	33.1 (1,198/3,617)	34.7 (1,433/4,134)	0.153

*The red number highlights the p which was less than 0.05 signifing the statistic difference*.

Table 3 shows the oocyte retrieval cycle characteristics of patients in the OMA group and the peritoneal EMS group. Comparing with the peritoneal EMS group, the OMA group had a higher percentage of oocytes that were inseminated by ICSI (17.7 vs. 10.5%, *p* < 0.001). With a higher percentage of POR and a lower oocyte retrieval rate, the number of follicles with diameter >14 mm and number of oocytes retrieved were significantly less in the OMA group compared with those of the peritoneal EMS group (all *p* < 0.05; Table 3). However, mature oocyte rate, fertilization rate, normal fertilization rate, viable embryo rate per oocyte retrieved, and high-quality embryo rate per oocyte retrieved were significantly higher in the OMA group comparing with the peritoneal EMS group (all *p* < 0.01).

**Table 3:** Oocyte retrieval cycle characteristics of the patients in the OMA group and peritoneal EMS group.

**Characteristics**	**OMA group**	**Peritoneal EMS group**	***p***
No. of patients	363	96	
No. of oocyte retrieval cycles	363	96	
No. of >14 mm follicles on the trigger day	6.79 ± 5.67	8.21 ± 5.50	0.028
No. of oocytes retrieved, *n*	7.50 ± 6.07	9.30 ± 6.47	0.011
No. of MII oocytes retrieved, *n*	6.32 ± 5.25	7.36 ± 5.58	0.087
Method of insemination			0.000
IVF, %	82.3(2,028/2,465)	89.5(722/807)	
ICSI, %	17.7(437/2,465)	10.5(85/807)	
No. of D3 top-quality embryos, *n*	2.60 ± 2.71	2.66 ± 2.78	0.852
No. of embryos cultured to blastocyst stage No. of viable blastocyst Viable blastocyst rate, %	1,029 177 17.2	305 45 14.8	0.314
No. of viable embryos, *n*	2.93 ± 2.64	2.94 ± 2.72	0.983
Oocyte retrieval rate, %	67.8 (2,724/4,016)	72.4 (893/1,234)	0.003
Poor ovarian response, %	21.2 (77/363)	11.5 (11/96)	0.031
Mature oocyte rate, %	84.2 (2,294/2,724)	79.2 (707/893)	0.001
Fertilization rate, %	85.3 (2,102/2,465)	75.6 (610/807)	0.000
Normal fertilization rate, %	80.6 (1,986/2,465)	70.1 (566/807)	0.000
Cleavage rate, %	97.1 (1,929/1,986)	96.8 (548/566)	0.700
Viable embryo rate per oocyte retrieved, %	39.1 (1,064/2,724)	31.6 (282/893)	0.000
High-quality embryo rate per oocyte retrieved, %	34.6 (943/2,724)	28.6 (255/893)	0.001

*The red number highlights the p which was less than 0.05 signifing the statistic difference*.

Table 4 shows the oocyte retrieval cycle characteristics of patients in the cystectomy group and the aspiration group. The aspiration group had higher proportion of oocytes inseminated by ICSI comparing with the cystectomy group (29.2 vs. 8.8%, *p* < 0.001). In the comparison between the two groups, the patients who underwent cystectomy had significantly fewer follicles with diameter of >14 mm, oocytes retrieved, MII oocytes retrieved, and viable embryos compared with those with intact endometrioma (all *p* < 0.05).

**Table 4:** Oocyte retrieval cycle characteristics of the patients in the cystectomy group and aspiration group.

**Characteristics**	**Cystectomy group**	**Aspiration group**	***p***
No. of patients	224	139	
No. of oocyte retrieval cycles	224	139	
No. of >14 mm follicles on the trigger day	6.13 ± 5.34	7.85 ± 6.02	0.005
No. of oocytes retrieved, *n*	6.77 ± 5.76	8.69 ± 6.38	0.003
No. of MII oocytes retrieved, *n*	5.70 ± 4.87	7.34 ± 5.68	0.004
Method of insemination			0.000
IVF, %	91.2 (1,265/1,387)	70.8 (763/1,078)	
ICSI, %	8.8 (122/1,387)	29.2 (315/1,078)	
No. of D3 top-quality embryos, *n*	2.42 ± 2.60	2.88 ± 2.86	0.120
No. of embryos cultured to blastocyst stage No. of viable blastocyst Viable blastocyst rate, %	583 89 15.3	446 88 19.7	0.060
No. of viable embryos, *n*	2.67 ± 2.44	3.36 ± 2.89	0.019
Oocyte retrieval rate, %	68.3 (1,516/2,220)	67.3 (1,208/1,796)	0.488
Poor ovarian response, %	23.2 (52/224)	18.0 (25/139)	0.236
Mature oocyte rate, %	84.1 (1,275/1,516)	84.4 (1,019/1,208)	0.858
Fertilization rate, %	86.2 (1,196/1,387)	84.0 (906/1,078)	0.129
Normal fertilization rate, %	81.5 (1,130/1,387)	79.4 (856/1,078)	0.199
Cleavage rate, %	97.1 (1,097/1,130)	97.2 (832/856)	0.877
Viable embryo rate per oocyte retrieved, %	39.4 (597/1,516)	38.7 (467/1,208)	0.702
High-quality embryo rate per oocyte retrieved, %	35.8 (542/1,516)	33.2 (401/1,208)	0.164

*The red number highlights the p which was less than 0.05 signifing the statistic difference*.

### Outcomes in FET Cycles

Frozen–thawed embryo transfer outcomes are presented in Table 5. The cumulative clinical pregnancy rate was 56.9% (238/418) in the EMS group, in contrast with 75.3% (244/324) in the control group (*p* < 0.001). In other two comparisons, no significant difference was found in all characteristics of FET outcomes.

**Table 5:** Outcomes of FET in the groups.

**Characteristics**	**EMS group**	**Control group**	***p*1**	**Ovarian EMS group**	**Peritoneal EMS group**	***p*2**	**Cystectomy group**	**Aspiration group**	***p*3**
No. of patients	329	316		262	67		157	105	
No. of FET cycles	442	474		353	89		205	148	
No. of embryos transferred	806	888		643	163		372	271	
Endometrial thickness, mean ± SD	10.16 ± 4.39	9.54 ± 4.20	0.164	10.39 ± 4.26	9.24 ± 4.82	0.123	10.68 ± 3.97	9.95 ± 4.65	0.276
Clinical pregnancy rate per transfer, %	54.8 (242/442)	54.6 (259/474)	0.919	55.2 (195/353)	52.8 (47/89)	0.680	59.0 (121/205)	50.0 (74/148)	0.092
Implantation rate, %	38.1 (307/806)	36.6 (325/888)	0.526	38.3 (246/643)	37.4 (61/163)	0.845	40.3 (150/372)	35.4 (96/271)	0.207
Miscarriage rate, %	6.5 (20/307)	6.8 (22/325)	0.898	7.3 (18/246)	3.3 (2/61)	0.253	7.3 (11/150)	7.3 (7/96)	0.990
Ectopic pregnancy rate, %	0.9 (4/442)	2.1 (10/474)	0.138	1.1 (4/353)	0 (0/89)	0.588	1.5 (3/205)	0.7 (1/148)	0.642
Cumulative clinical pregnancy, %	56.9 (238/418)	75.3 (244/324)	0.000	57.4 (191/333)	55.3 (47/85)	0.732	57.6 (118/205)	57.0 (73/128)	0.967
Live birth rate per transfer, % Term delivery, *n* Preterm delivery, *n*	47.5 (210/442) 173 37	45.8 (217/474) 176 41	0.600	47.3 (167/353) 137 30	48.3 (43/89) 36 7	0.865	48.8 (100/205) 85 15	45.3 (67/148) 52 15	0.515
Birth weight (g), mean ± SD	3,009 ± 682	2,987 ± 607	0.682	2,993 ± 689	3,074 ± 654	0.444	3,018 ± 644	2,957 ± 755	0.526

*p1, EMS group vs. control group; p2, ovarian EMS group vs. peritoneal EMS group; p3, cystectomy group vs. aspiration group. The red number highlights the p which was less than 0.05 signifing the statistic difference*.

### Establishment of Predictive Model of Cumulative Clinical Pregnancy

To evaluate the relative predictive value of group allocation, age, duration of infertility, BMI (D3) FSH, LH, E_2_, AFC, number of oocytes retrieved, POR, oocyte retrieval rate, mature oocyte rate, and fertilization rate for cumulative clinical pregnancy, which was defined as having a history of clinical pregnancy by transferring embryos from this oocyte retrieval cycle, binary logistic regression analysis was performed in respective population of each comparison.

Antral follicle count was directly associated with cumulative clinical pregnancy [odds ratio (OR) 1.054; 95% confidence interval (CI), 1.011–1.100; *p* = 0.014] in the population of the EMS and the control. E_2_ (D3) level was inversely associated with cumulative clinical pregnancy in the OMA population (OR, 0.984; 95% CI, 0.970–0.998; *p* = 0.028) and the entire EMS population (OR, 0.988; 95% CI, 0.977–1.000; *p* = 0.047; Table 6).

**Table 6:** Logistic regression of cumulative clinical pregnancy.

**Characteristics**	**EMS group vs. control group**			**OMA group vs. peritoneal EMS group**			**Cystectomy group vs. aspiration group**		
	**OR**	***p***	**95% CI**	**OR**	***p***	**95% CI**	**OR**	***p***	**95% CI**
Group	1.224	0.301	0.835–1.794	1.355	0.360	0.707–2.598	1.400	0.274	0.766–2.559
Age, y	0.970	0.284	0.918–1.025	0.965	0.384	0.891–1.046	0.974	0.578	0.889–1.068
Duration of infertility, y	0.980	0.595	0.910–1.055	0.962	0.524	0.854–1.083	0.956	0.538	0.829–1.103
BMI (kg/m^2^)	0.957	0.343	0.874–1.048	0.957	0.445	0.854–1.072	0.915	0.160	0.807–1.036
FSH (IU/L)	0.928	0.100	0.849–1.014	0.908	0.073	0.816–1.009	0.918	0.134	0.820–1.027
LH (IU/L)	1.062	0.338	0.940–1.199	1.119	0.221	0.935–1.340	1.142	0.190	0.936–1.393
E_2_ (pg/mL)	0.992	0.058	0.984–1.000	0.988	0.047	0.977–1.000	0.984	0.028	0.970–0.998
AFC	1.054	0.014	1.011–1.100	1.031	0.269	0.977–1.088	1.003	0.946	0.929–1.082
No. of oocytes retrieved, *n*	1.015	0.386	0.981–1.051	1.031	0.253	0.978–1.087	1.051	0.148	0.983–1.124
POR	1.390	0.462	0.578–3.344	1.790	0.277	0.627–5.113	1.761	0.329	0.566–5.483
Oocyte retrieval rate, %	1.531	0.208	0.789–2.973	1.329	0.566	0.503–3.513	0.627	0.424	0.200–1.966
Mature oocyte rate, %	0.565	0.327	0.180–1.772	0.480	0.357	0.101–2.289	0.493	0.439	0.082–2.958
Fertilization rate, %	0.788	0.674	0.259–2.392	0.869	0.852	0.200–3.774	0.421	0.317	0.077–2.294

*OR, odds ratio; BMI, body mass index; FSH, follicle stimulating hormone; LH, luteinizing hormone; E_2_,estrogen; AFC, antral follicle count; hMG, human menopausal gonadotropin; MPA, medroxyprogesterone acetate. The red number highlights the p which was less than 0.05 signifing the statistic difference*.

**Figure 2:**
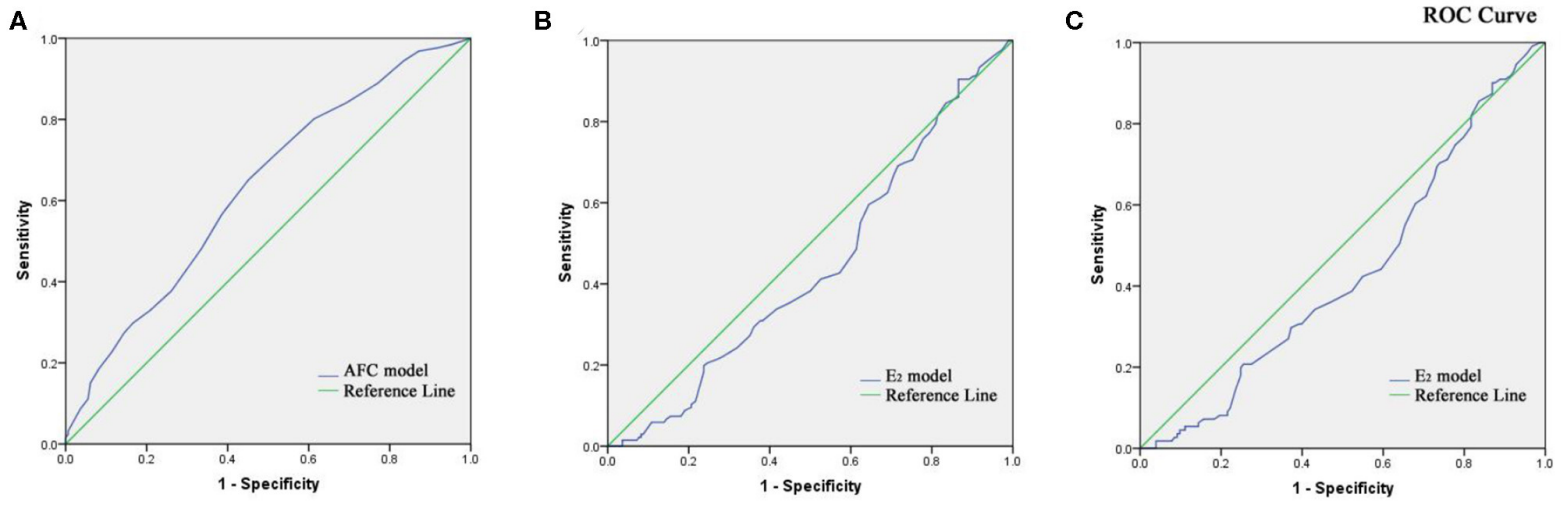
**(A)** Receiver operating characteristic curve of the AFC prediction model of cumulative clinical pregnancy in the population of the EMS and the control. **(B)** Receiver operating characteristic curve of the E_2_ prediction model of cumulative clinical pregnancy in the population of EMS. **(C)** Receiver operating characteristic curve of the E_2_ prediction model of cumulative clinical pregnancy in the population of OMA.

The two key factors and their regression coefficients were used to establish probability models for predicting cumulative clinical pregnancy in respective populations. Figure 2A presents the corresponding receiver operating characteristic (ROC) curve for the AFC prediction model of cumulative clinical pregnancy in the population of the EMS and the control, with an area under the curve (AUC) of 0.60 (95% CI, 0.57–0.64; *p* < 0.001). Figures 2B,C present the corresponding ROC curve for the E_2_ prediction model of cumulative clinical pregnancy in the entire EMS population and the OMA population, respectively, both with no significance.

## Discussion

For over two decades, the detrimental effect of EMS on ART pregnancy outcomes has been disputed in the literature. We reviewed the published cohort–control studies (retrospective or prospective) and randomized controlled trials since 2000. The results of specific parameters in these studies were categorized according to the characteristics that were found significantly different in groups in this study (Table 7).

**Table 7:** Outcomes of IVF/ICSI-FET in studies about EMS.

**Study**	**Endometriosis**	**No endometriosis**	***p***
**Primary infertility**, ***n*** **(%)**			
Bourdon et al. (5)	161 (80.0)	283 (70.4)	<0.001
Benaglia et al. (24)	34 (87.0)	62 (80.0)	NS
Nicolaus et al. (25)	41 (87.2)	128 (81.0)	NS
Ashrafi et al. (26)	116 (80.0)	128 (97.7)	<0.001
**Duration of infertility, y (mean** **±** **SD)**			
Benaglia et al. (27)	3.4 ±.0	4.0 ± 2.4	0.002
Nicolaus et al. (25)	3.67 ± 2.65	2.91 ± 2.04	NS
Ashrafi et al. (26)	6.3 ± 3.6	5.8 ± 2.5	NS
**AFC (mean** **±** **SD)**			
Bourdon et al. (5)	13.1 ± 7.4	15.5 ± 10.1	0.003
Reinblatt et al. (28)	14.8 ± 4.5	21.5 ± 3.3	NS
Benaglia et al. (24)	11 ± 1	12 ± 8	NS
Luca et al. (29)	12.54 ± 5.26	13.39 ± 5.86	NS
Nicolaus et al. (25)	11.80 ± 5.1	10.90 ± 5.6	NS
Ashrafi et al. (26)	8.2 ± 3.9	12.0 ± 4.8	<0.001
**No. of oocytes retrieved (mean** **±** **SD)**			
Bourdon et al. (5)	7.5 ± 5.4	9.4 ± 6.1	<0.001
Al-Azemi et al. (30)	6.9 ± 0.7	7.1 ± 0.5	NS
Omland et al. (31)	9.4 ± 4.9	10.0 ± 5.6	NS
Fernandez et al. (32)	10.5	12.8	NS
Reinblatt et al. (28)	11.2 ± 1.6	12.3 ± 1.05	NS
Rajani et al. (33)	5.54 ± 2.8	5.27 ± 3.4	NS
Benaglia et al. (24)	7.1 ± 3.2	9.8 ± 5.5	NS
Benaglia et al. (27)	7.0 ± 4.0	7.9 ± 4.3	0.04
Chauffour et al. (6)	8.37 ± 7.01	10.13 ± 6.53	<0.001
Luca et al. (29)	10.50 ± 4.74	9.02 ± 4.53	NS
Yamamoto et al. (34)	11.87 ± 6.29	12.71 ± 7.33	NS
Feichtinger et al. (7)	8.47	9.54	0.0146
Nicolaus et al. (25)	5.89	7.25	0.045
**POR**, ***n*** **(%)**			
Bourdon et al. (5)	62 (30.8)	90 (22.3)	0.02
**No. of MII oocytes retrieved (mean** **±** **SD)**			
Bourdon et al. (5)	6.4 ± 4.8	7.6 ± 5.0	0.009
Fernandez et al. (32)	7.1	9.4	NS
Reinblatt et al. (28)	9.5 ± 1.7	13.2 ± 0.96	NS
Benaglia et al. (24)	5.1 ± 2.5	6.9 ± 4.2	NS
Luca et al. (29)	8.79 ± 4.65	8.23 ± 4.06	NS
Nicolaus et al. (25)	4.87	6.04	0.046
**Fertilization rate (%)**			
Nicolaus et al. (25)	40.61	57.76	0.003
Reinblatt et al. (28)	66	73	NS
Rajani et al. (33)	81.4	81.4	NS
Omland et al. (31)	68.7	68.7	NS
Bourdon et al. (5)	70	70	NS
**No. of viable embryos (mean** **±** **SD)**			
Bourdon et al. (5)	4.4 ± 3.4	4.9 ± 3.7	NS
Benaglia et al. (24)	2.6 ± 1.4	3.1 ± 1.5	NS
Chauffour et al. (6)	4.81 ± 4.72	5.01 ± 4.39	NS
Feichtinger et al. (7)	4.35	4.32	NS
**Clinical pregnancy rate per transfer (%)**			
Bourdon et al. (5)	35	41.3	NS
Fernandez et al. (32)	34.6	39.4	NS
Mitwally et al. (35)	41.6	51.0	NS
Rajani et al. (33)	32.1	43.75	NS
Benaglia et al. (24)	39	37	NS
Turocy et al. (36)	50.6	47.8	NS
Feichtinger et al. (7)	35.8	34.55	NS
**Live birth per transfer (%)**			
Bourdon et al. (5)	39 (25.8)	99 (30.5)	NS
Omland et al. (31)	140 (66.0)	360 (66.7)	NS
Benaglia et al. (24)	29	33	NS
Chauffour et al. (6)	30.3	28.8	NS
Turocy et al. (36)	32 (44.4%)	533 (41.1%)	NS
**Cumulative clinical pregnancy rate per oocyte retrieval (%)**			
Bourdon et al. (5)	45	51.5	NS
Fernandez et al. (32)	33	34.9	NS
Benaglia et al. (24)	33	35	NS
Feichtinger et al. (7)	42.61	40.41	NS

*NS, no significance*.

The outcome parameters evaluated were mostly clinical pregnancy rate and live birth rate, whereas cumulative clinical pregnancy rate was evaluated only in a few reports. No significant difference was found in the outcome parameters in these reports; however, some characteristics in oocyte retrieval cycle were found significantly different between EMS and non-EMS, especially the number of oocytes retrieved. With the same clinical pregnancy rate per frozen embryos transfer, the more embryos that could be transferred, the higher cumulative clinical pregnancy rate of this oocyte retrieval cycle. The significant decrease in the number of oocytes retrieved in EMS was not positive correlation with the number of viable embryos (1, 13), which may be the reason for no significant difference in cumulative clinical pregnancy rate. After all, it cannot be considered that EMS increases oocyte fertilization rate. In this study, with a large sample, cumulative clinical pregnancy was found significantly decreased in the patients with advanced EMS compared with the control. The lower cumulative clinical pregnancy rate accompanied by normal clinical pregnancy rate per transfer occurs only in fewer FET cycles, which was further evidenced by a lower average FET cycle per patient in the EMS group (1.34 in the EMS group, 1.5 in the control group). We hypothesize that fewer oocytes retrieved, followed by less frozen embryos, could be the main cause for the less FET cycles in the EMS group compared with the control group.

The number of oocytes retrieved depends on ovarian response to stimulation. In the Bologna criteria (21), retrieved oocytes of three or fewer are a direct indicator of POR, which was not a parameter in oocyte retrieval cycle in most previous studies. Another minimal criterion diagnosing POR is an abnormal ovarian reserve test, which can be represented by the reduced AFC. In this study, AFC was found to be an independent factor associated with cumulative clinical pregnancy in an oocyte retrieval cycle, and the AFC prediction model was with an AUC of 0.6. Follicle-stimulating hormone is also a factor to assess ovarian reserve (37), whose level corresponds with the decline in oocyte quantity (38). In this study, the positive correlation between baseline FSH level and POR was found in the three comparisons. Similar to the results of this study, Coelho Neto et al. (39) found a higher proportion of POR in patients with EMS, but the chance of achieving live birth was similar between women with EMS and those without it (19.1 vs. 22.5%) and also when considering only women with a POR (9.4 vs. 8.9%) and only those with a normal ovarian reserve (25.5 vs. 26.5%). Poor ovarian response is often associated with diminished ovarian reserve (DOR) (40), which resulted in low conception rates and high rates of fetal loss in earlier studies (41). Diminished ovarian reserve is diagnosed as the reduced capacity of the ovaries to produce oocytes in both quantity and quality (42). The patients with advanced EMS had significantly lower viable embryo rate per oocyte retrieved and viable blastocyst rate, which indicated the lack of competence in oocytes. Combined with the deficiency in quantity and quality of oocytes found in this study, DOR may be one of the critical factors of negative effect of advanced EMS on poor IVF/ICSI-FET outcomes.

In the previous studies, the difference in IVF-ET outcomes between the patients with a history of OMA and the patients without EMS was found, whereas there were few data about peritoneal EMS. To our knowledge, at present, there is no study with large sample about the difference of IVF/ICSI-FET outcomes between OMA and peritoneal EMS. Outside ovaries, is there any negative effect of EMS on IVF/ICSI-FET outcomes? Because OMA and DIE are highly coexisting (43), it is hard to include the cases of OMA without peritoneal ectopic lesions in the clinic. In the same scoring system of the rAFS classification, OMAs account for most of the scores in OMA patients, and patients with peritoneal EMS have more ectopic lesions, which suggest greater effect of factors outside the ovaries. The OMA group had poorer ovarian response than the peritoneal EMS group; however, oocyte competence to maturation in the OMA group appeared to be higher than that of the peritoneal EMS group in this study according to the significant difference in mature oocyte rate. The method of insemination should be considered as a factor that may affect fertilization; therefore, the oocyte competence to fertilization in the peritoneal EMS group could not be determined, although with lower fertilization rate, normal fertilization rate, viable embryo rate, and high-quality embryo rate per oocyte retrieved comparing with the OMA group. Ashrafi et al. (26) found the existence of DIE was associated with a significant decrease in AFC and ovarian sensitivity index, which was computed as the total number of retrieved oocytes divided by the total dose of recombinant FSH administered, whether with OMA or not. How does DIE affect ovarian reserve and ovarian stimulation outcomes? The changed intraperitoneal and follicular microenvironment of patients with EMS has been widely reported. The excessive reactive oxygen species generation was observed in both peritoneal fluid and follicular fluid of EMS (44, 45), which could impair meiotic spindles, resulting in the inability of oocytes to complete maturation (46, 47) and increase in DNA damage in granulosa cells (48). In the follicular fluid in patients with EMS, the concentration of inflammatory cytokines, including interleukin 1b (IL-1b), IL-8, and IL-10 and tumor necrosis factor, shows distinctive patterns and increased levels, which may be linked to reduced ovarian response (49). Although chronic inflammation is commonly thought to be a feature of EMS (50), OMA seldom induces inflammation in nearby follicles during IVF (49). We hypothesize that, combined with the results of this study, endometriomas do not directly influence folliculogenesis and oocyte competence. Santulli et al. (10) also demonstrated that OMA *per se* is not associated with presentation for infertility, and peritoneal superficial EMS is a risk factor for EMS-related infertility. In addition, those cytokines associated with folliculogenesis and oocyte maturation were found to be less expressed in follicular fluid of EMS, such as retinoids (51), brain-derived neurotrophic factor (52), and growth differentiation factor 9 (53). However, the mechanisms behind these changes in follicular fluid of EMS, resulting in disturbed folliculogenesis and poor-quality oocytes, are not clear yet.

Both OMA *per se* and its surgical removal may directly impair ovarian reserve. The endometriotic tissue in OMA may secrete a number of products, including cytokines, chemokines, and growth factors, which may activate specific signaling pathways in the follicular cells, leading to premature follicular development and accelerated atresia (9, 54). Some reports suggested that surgical treatment to OMA may improve spontaneous pregnancy rates by restoring the peritoneal anatomy (14, 55), and its detrimental effects on ovarian reserve appears to temporary (13). For patients going into assisted reproductive treatment, the explicit benefits from surgery to pregnancy outcomes in IVF/ICSI are important. Surgical excision may have a greater impact on ovarian response according to the results of this study. Although there was no significant difference in AFC and the proportion of POR, the number of dominant follicles, number of oocytes retrieved, number of mature oocytes retrieved, and number of viable embryos in the cystectomy group were significantly fewer than those of the aspiration group, similar to the results of Tao et al. (56). The number of viable embryos determines the chances of transfer, and its reduction means lower probability of pregnancy. However, the patients who had undergone surgery had no better pregnancy outcomes in IVF/ICSI than those of patients with the presence of OMA. No significant benefits of surgical treatment prior to IVF/ICSI were found in this study.

In conclusion, advanced EMS has negative effect on IVF/ICSI-FET outcomes, which is reflected on the cumulative clinical pregnancy per oocyte retrieval cycle, and AFC is an independent predictor. The negative effect is mainly caused by POR associated with OMA *per se* or its surgery and the damage of peritoneal EMS to oocyte maturation. The surgical excision of OMA prior to IVF/ICSI would not improve FET outcomes.

## Data Availability Statement

All datasets generated for this study are included in the article/supplementary material.

## Ethics Statement

The studies involving human participants were reviewed and approved by the Ethics Committee (Institutional Review Board) of the Ninth People's Hospital of Shanghai. The patients/participants provided their written informed consent to participate in this study. Written informed consent was obtained from the individual(s) for the publication of any potentially identifiable images or data included in this article.

## Author Contributions

CY and YK supervised the entire study, including the procedures, conception, design and completion, participated in the interpretation of the study data, and in revisions to the article. JZ was responsible for the collection of data. AL contributed the data analysis and drafted the article. All authors contributed to the article and approved the submitted version.

## Conflict of Interest

The authors declare that the research was conducted in the absence of any commercial or financial relationships that could be construed as a potential conflict of interest.

## References

[1] Bulun SE Endometriosis. N Engl J Med. (2009) 3:268–79. 10.1056/NEJMra0804690 19144942

[2] Dunselman GA Vermeulen N Becker C Calhaz-Jorge C D'Hooghe T de Bie B. European Society of Human, and Embryol, ESHRE guideline: management of women with endometriosis. Hum Reprod. (2014) 29:400–12. 10.1093/humrep/det457 24435778

[3] Bulletti C Coccia ME Battistoni S Borini A. Endometriosis and infertility. J Assist Reprod Genet. (2010) 27:441–7. 10.1007/s10815-010-9436-1 20574791 PMC2941592

[4] Kuivasaari P Hippelainen M Anttila M Heinonen S. Effect of endometriosis on IVF/ICSI outcome: age III/IV endometriosis worsens cumulative pregnancy and live-born rates. Hum Reprod. (2005) 20:3130–5. 10.1093/humrep/dei176 16006468

[5] Bourdon M Raad J Dahan Y Marcellin L Maignien C Even M. Endometriosis and ART: a prior history of surgery for OMA is associated with poor ovarian response to hyperstimulation. PLoS ONE. (2018) 8:1–16. 10.1371/journal.pone.0202399 30125306 PMC6101383

[6] Chauffour C Pouly JL Brugnon F Dejou L Gremeau AS Janny L Effect of endometriosis on IVF outcomes in cases of single embryo transfer for first IVF attempt in pents under 35. J Endometr. (2016) 8:13–8. 10.5301/je.5000230

[7] Feichtinger M Nordenhok E Olofsson JI Hadziosmanovic N Rodriguez-Wallberg KA. Endometriosis and cumulative live birth e after fresh and frozen IVF cycles with single embryo transfer in young women: no impact beyond reduced ovarian sensitivity-a case control study. J Assist Reprod Genet. (2019) 36:1649–56. 10.1007/s10815-019-01519-5 31313013 PMC6707971

[8] Tosti C Pinzauti S Santulli P Chapron C Petraglia F. Pathogenetic mechanisms of deep infiltrating endometriosis. Reprod Sci. (20122:1053–9. 10.1177/1933719115592713 26169038

[9] Bulun SE. Ovarian endometriosis: the nemesis of eggs. Fertil Steril. (2014) 101:938–9. 10.1016/j.fertnstert.2014.01.044 24613535 PMC3972349

[10] Santulli P Lamau MC Marcellin L Gayet V Marzouk P Borghese B. Endometriosis-related infertility: ovarian endometrioma per se is not associated withesentation for infertility. Hum Reprod. (2016) 31:1765–75. 10.1093/humrep/dew093 27130614

[11] Alborzi S Keramati P Younesi M Samsami A Dadras N The impact of laparoscopic cystectomy on ovarian reserve in patients with unilateral and bilateral endometriomas. Fertil Steril. (201401:427–34. 10.1016/j.fertnstert.2013.10.019 24269044

[12] Carneiro MM Costa LMP Avila I To operate or not to operate on women with deep infiltrating endometriosis (DIE) before *in vitro* fertilization (IVF). JBRA Assist Reprod. (2017) 21:120–5. 10.5935/1518-0557.20170027 28609279 PMC5473705

[13] Goodman LR Goldberg JM Flyckt RL GuM Harwalker J Falcone T. Effect of surgery on ovarian reserve in women with endometriomas, endometriosis and controls. Am J Obstet Gynecol. (2016) 215:589.e1–6. 10.1016/j.ajog.2016.05.029 27242204

[14] Raffi F Amer SA. Long-term reproductive performance after surgery for ovarian endometrioma. Eur J Obstet Gynecol Reprod Biol. (2014) 172:80–4. 10.1016/j.ejogrb.2013.09.042 24231199

[15] Guinard E Collinet P Lefebvre C Robin G Rubod C. Management of infertile women with pelvic endometriosis: a literature review. Minerva Ginecol. (2017) 69:178–89. 10.23736/S0026-4784.16.03989-7 27905697

[16] Fan YY Chen HY Chen W Liu YN Fu Y Wang LN. Expression of inflammatory cytokines in serum and perito fluid from patients with different stages of endometriosis. Gynecol Endocrinol. (2018) 34:507–12. 10.1080/09513590.2017.1409717 29308924

[17] Zhang QF Chen GY Liu Y Huang HJ Song YF. Relationship between resistin IL-23 levels in follicular fluid in infertile patients with endometriosis undergoing IVF-ET. Adv Exp Med. (2017) 26:1431–35. 10.17219/acem/41149 29442466

[18] Malvezzi H Da Broi MG Meola J Rosa SJC Ferriani RA Navarro PA. Peritoneal fluid of women with endometriosis reduces SOD1 in bovine oocytes *in vitro* maturation. Cell Tissue Res. (2018) 372:621–28. 10.1007/s00441-018-2805-2 29464366

[19] Cummins JM Breen TM Harri KL Shaw JM Wilson LM Hennessey JF. A formula for scoring human embryo growth rates in *in vitro* fertilization: its value in predicting pregnancy and in comparison with visual estimates of embryo quality. J In vitro Fert Embryo Transf. (1986) 84–95. 10.1007/BF01133388 3783014

[20] Yu S Long H Chang HY Liu Y Gao H Zhu J. New application of dydrogesterone as a part of a progestin-primed ovarian stimulation protocol for IVF: a randomized controlled trial including 516 first IVF/ICSI cycles. Hum Reprod. (2018) 33:229–37. 10.1093/humrep/dex367 29300975

[21] Ferraretti AP La Marca A Fauser BC Tarlatzis B Gund G Gianaroli L. ESHRE consensus on the definition of 'poor response' to ovarian stimulation for *in vitro* fertilization: the Bologna criteria. Hum Reprod. (2011) 26:1616–24. 10.1093/humrep/der092 21505041

[22] Calhaz-Jorge C De Geyter C Kupka MS de Mouzon J Erb K Mocanu E. Assisted reproductive technology in Europe, 2013: results generated from European registers by ESHRE. Hum Reprod. (2017) 32:1957–73. 10.1093/humrep/dex264 29117383

[23] Ham M Omar SZ Dunselman G Cheong Y Influence of endometriosis on assisted reproductive technology outcomes: a systematic review and meta-analysis. Obstetr. Gynecol. (2015) 1:79–88. 10.1097/AOG.0000000000000592 25560108

[24] Benaglia L Bermejo A Somigliana E Faulisi S Ragni G Fedele L. *In vitro* fertilization outcome in women with unoperated bilateral endometriomas. Fertil Steril. (2013) 99:1714–9. 10.1016/j.fertnstert.2013.01.110 23415975

[25] Nicolaus K Brauer D Sczesny R Jimenez-Cruz J Buhler K Hoppe I. Endometriosis reduces ovarian response in controlled ovarian hyperstimulation independent of AMH, AFC, and women's age measured by follicular output rate (FORT) and number of oocytes retrieved. Arch Gynecol Obstet. (2019) 300:1759–65. 10.1007/s00404-019-05337-z 31667607

[26] Ashrafi M Arabipoor A Hemat M Salman-Yazdi R. The impact of the localisation of endometriosis lesions on ovarian reserve and assisted reproduction techniques outcome Obstet Gynaecol. (2019) 39:91–97. 10.1080/01443615.2018.1465898 30257599

[27] Benaglia L Candotti G Papaleo E Pagliardini L Leonardi M Reschini M. Pregnancy outcome in women with endometriosis achieving pregnancy with IVF. Hum Reprod. (2016) 31:2730–36. 10.1093/humrep/dew210 27664955

[28] Reinblatt SL Ishai L Shehata F Son WY Tulandi T Almog B. Effects of ovarian endometrioma on embryo quality. Fertil Steril. (2011) 95:2700–2. 10.1016/j.fertnstert.2011.03.002 21444070

[29] Luca A Nemescu D Butnaru M Butnariu A Onofriescu M. Ovarian stimulation outcome in infertile women with endometriosis undergoing IVF. Ginekol Pol. (2016) 87:37–41. 10.17772/gp/60073 27306467

[30] Al-Azemi M Bernal AL Steele J Gramsbergen I Barlow D Kennedy S. Ovarian response to repeated controlled stimulation in in-vitro fertilization cycles in patients with ovarian endometriosis. Hum Reprod. (2000) 1:72–5. 10.1093/humrep/15.1.72 10611191

[31] Omland AK Abyholm T Fedorcsak P Ertzeid G Oldereid NB Bjercke S. Pregnancy outcome after IVF and ICSI in unexplained, endometriosis-associated and tubal factor infertility. Hum Reprod. (2005) 20:722–7. 10.1093/humrep/deh664 15591078

[32] Fernandez M Sanabria V Chinea E Hernandez J Palumbo A P-359: comparison of *in vitro* fertilization (IVF) outcome in patients with endometriosis and patients with other infertility causes. Fertil Steril. (2006) 86:S268–9. 10.1016/j.fertnstert.2006.07.71517055839

[33] Rajani S Sharma S Chakravarty B Chattopadhyay R Ghosh S Goswami S. Assessment of oocyte quality in polycystic ovarian syndrome and endometriosis by spindle imaging and reactive oxygen species levels in follicular fluid and its relationship with IVF-ET outcome. J Hum Reprod Sci. (2012) 5:187. 10.4103/0974-1208.101020 23162358 PMC3493834

[34] Yamamoto A Johnstone EB Bloom MS Huddleston HG Fujimoto VY. A higher prevalence of endometriosis among Asian women does not contribute to poorer IVF outcomes. J Assist Reprod Genet. (2017) 34:765–74. 10.1007/s10815-017-0919-1 28417349 PMC5445055

[35] Mitwally MF Imam M Fakih M Ashraf M Diamond MP Abuzeid M Diagnosis of polycystic ovarian syndrome (PCOS) or endometriosis is associated with lower pregnancy rates in women undergoing *in vitro* fertilization and embryo transfer (IVF-ET). Fertil Steril. (2007) 88:S120 10.1016/j.fertnstert.2007.07.386

[36] Turocy J Farland LV Yanushpolsky E Pregnancy outcomes in frozen embryo transfers in women with endometriosis: a retrospective cohort study. Fertil Steril. (2017) 108:e198 10.1016/j.fertnstert.2017.07.584

[37] Yakubu MT Olutoye AF. Aphrodisiac activity of aqueous extract of *Anthonotha macrophylla* P. Beauv. leaves in female Wistar rats. J Integr Med. (2016) 14:400–08. 10.1016/S2095-4964(16)60271-6 27641611

[38] Bishop LA Richter KS Patounakis G Andri L Moon K Devine K Diminished ovarian reserve as measured by means of baseline follicle-stimulating hormone and antral follicle count is not associated with pregnancy loss in younger *in vitro* fertilization patients. Fertil Steril. (2017) 108:980–87. 10.1016/j.fertnstert.2017.09.011 29202975

[39] Coelho Neto MA de Paula Martins W da Luz CM Melo BTG Ferriani RA Navarro PA. Endometriosis ovarian reserve and live birth rate following *in vitro* fertilization/intracytoplasmic sperm injection. Rev Bras Ginecol Obstet. (2016) 38:218–24. 10.1055/s-0036-1584126 27196950 PMC10309427

[40] Yun BH Kim G Park SH Noe EB Seo SK Cho S. *In vitro* fertilization outcome in women with diminished ovarian reserve. Obstetr Gynecol Sci. (2017) 60:46. 10.5468/ogs.2017.60.1.46 28217671 PMC5313363

[41] Levi AJ Raynault MF Bergh PA Drews MR Miller BT Scott RT Reproductive outcome in patienith diminished ovarian reserve. Fertil Steril. (2001) 4:666–69. 10.1016/S0015-0282(01)02017-9 11591396

[42] Zangmo R Singh N Sharma JB Diminished ovarian reserve and premature ovarian failure: a review. IVF Lite. (2016) 3:46 10.4103/2348-2907.192284

[43] Reid S Condou G Endometriomas and pelvic endometriosis. In: Guerriero S, Martins W, Alcazar J, editors. Managing Ultrasonography in Human Reproduction. Cham: Springer (2017). p. 123–36. 10.1007/978-3-319-41037-1_7

[44] Da Broi MG Andrade AZ Rodrigues JK de Paz CCP Jordão AA Navarro PA Total antioxidant capacity (TAC) levels in follicular fluid of infertile patients undergoing ICSI: a possible predr of clinical pregnancy. Fertil Steril. (2013) 100:S425 10.1016/j.fertnstert.2013.07.581

[45] Polak G Wertel I Barczynski B Kwaśniewski W Bednarek W Kotarski J. Increased levels of oxidative stress markers in the peritoneal fluid of women with endometriosis. Eur J Obsics Gynecol Reprod Biol. (2013) 168:187–90. 10.1016/j.ejogrb.2012.12.043 23351670

[46] da Broi MG Navarro PA. Oxidative stress and oocyte quality: ethiopathogenic mechanisms of minimal/mild metriosis-related infertility. Cell Tissue Res. (2015) 364:1–7. 10.1007/s00441-015-2339-9 26685866

[47] Da Broi M.G. Malvezzi H Paz CC Ferriani RA Navarro PA. Follicular fluid from infertile women with mild endometriosis may compromise the meiotic spindles of bovine metaphase II oocytes. Hum Reprod. (2014) 29:315–23. 10.1093/humrep/det378 24166595

[48] Singh AK Chattopadhyay R Chakravarty B Chaudhury K. Markers of oxidative stress in follicular fluid of women with endometriosis and tubal infertility undergoing IVF. Reprod Toxicol. (2013) 42:116–24. 10.1016/j.reprotox.2013.08.005 23994512

[49] Opoien HK Fedorcsak P Polec A Stensen MH Abyholm T Tanbo T. Do endometriomas induce an inflammatory reaction in nearby follicles? Hum Reprod. (2013) 28:1837–45. 10.1093/humrep/det087 23543385

[50] Tanbo T Fedorcsak P. Endometriosis-associated infertility: aspects of pathophysiological mechanisms and treatment options. Acta Obstet Gynecol Scand. (2017) 96:659–67. 10.1111/aogs.13082 27998009

[51] Pauli SA Session DR Shang W Easley K Wieser F Taylor RN. Analysis of follicular fluid retinoids in women undergoing *in vitro* fertilization: retinoic acid influences embryo quality and is reduced in women with endometriosis. Reprod Sci. (2013) 20:1116–24. 10.1177/1933719113477487 23427183 PMC3745715

[52] Zhang QY Guan Q Wang Y Feng X Sun W Kong FY. BDNF Val66Met polymorphism is associated with Stage III-IV endometriosis and poor *in vitro* fertilization outcome. Hum Reprod. (2012) 27:1668–75. 10.1093/humrep/des094 22447624

[53] Hendarto H Prabowo P Moeloek FA Soetjipto S. Growth differentiation factor 9 concentration in the follicular fluid of infertile women with endometriosis. Fertil Steril. (2010) 94:758–60. 10.1016/j.fertnstert.2009.10.011 19931079

[54] Oliver-Baxter JM Whitford HS Turnbull DA Bond MJ. Effects of vitamin supplementation on inflammatory markers and psychological wellbeing among distressed women: a randomized controlled trial. J Integr Med. (2018) 16:322–8. 10.1016/j.joim.2018.06.001 29929873

[55] Jayaprakasan K Becker C Mittal M The effect of surgery for endometriomas on fertility. BJOG. (2018) 125:e19–28. 10.1111/1471-0528.14834 28944556

[56] Tao X Chen L Ge S Cai L. Weigh the pros and cons to ovarian reserve before stripping ovarian endometriomas prior to IVF/ICSI: a meta-analysis. PLoS ONE. (2017) 12:e0177426. 10.1371/journal.pone.0177426 28574993 PMC5456033

